# Sporadic Hepatitis A Virus PCR False-Positive Results Observed during Reflex Testing of Serum Samples Previously Tested for Anti-HAV Antibodies and Caused by Contamination with HAV RNA Present in the Reagents of the Commercial Anti-HAV Immunoassay

**DOI:** 10.1128/spectrum.00122-23

**Published:** 2023-05-10

**Authors:** Maja M. Lunar, Petra Markočič, Kristina Fujs Komloš, Tina Štamol, Mario Poljak

**Affiliations:** a Institute of Microbiology and Immunology, Faculty of Medicine, University of Ljubljana, Ljubljana, Slovenia; University of Cincinnati

**Keywords:** Hepatitis A virus, HAV RNA, contamination, diagnostics, serology, reflex testing

## Abstract

Hepatitis A diagnosis relies on serology and occasionally on hepatitis A virus (HAV) RNA detection. For timely diagnosis and the avoidance of drawing additional blood, molecular testing is often performed as reflex testing by using blood specimens that were initially sent for anti-HAV serology. Reflex molecular testing is preferably performed from different sample aliquots, but, for limited sample quantities, it uses samples that have been preprocessed in an immunoassay analyzer. In 2012, we first observed sporadic HAV RNA-positive cases that were inconsistent with patients’ serological profiles and/or medical histories, suggesting that occasional laboratory contamination was causing false-positive PCR results. Multiple external quality assurance (EQA) and laboratory surface contamination checks were performed, questionable specimens were tested with various HAV RNA tests, and follow-up serum/stool samples were collected. All contamination-check samples and samples from healthy individuals tested HAV RNA-negative, and the laboratory successfully passed all EQAs. The HAV RNA-positive results were reproducible with various HAV RNA assays. No patients seroconverted, and their follow-up samples were consistently HAV RNA-negative. Finally, a detailed review of testing protocols revealed a correlation between HAV RNA false positivity and preceding anti-HAV testing with the Cobas-e411 automated immunoassay analyzer. HAV RNA was detected in the Cobas-e411 anti-HAV reagents, with the HAV sequences matching those from the false-positive samples. Preceding anti-HAV testing using two other immunoassay analyzers did not result in subsequent HAV RNA false positivity during reflex testing. The Cobas-e411 pipetting procedure with a single pipette tip collecting samples and anti-HAV reagents contaminated the original sample with the HAV RNA that was present in the immunoassay’s reagents, thereby resulting in HAV RNA false positivity during the reflex testing.

**IMPORTANCE** We present the first report of sporadic HAV PCR false-positive results that have been observed during the reflex testing of serum samples that have previously been tested for anti-HAV antibodies and have been caused by contamination with HAV RNA that is present in the reagents of the commercial anti-HAV immunoassay, with potentially serious clinical consequences. Although HAV RNA was consistently detected in the anti-HAV reagents of all three automated immunoassay analyzers that were in use in our laboratory, only the use of one analyzer and the corresponding commercial anti-HAV immunoassay reagents resulted in contamination that led to false positive HAV RNA results, and this was due to a peculiar pipetting mode of action in which the analyzer uses a single pipette tip to collect both anti-HAV reagents and a sample, which consequently causes the permanent contamination of the original sample with HAV RNA. Manufacturers should strongly consider the occasional need for reflex molecular testing from preprocessed samples and design their analyzers in a way that prevents contamination.

## INTRODUCTION

Hepatitis A virus (HAV) mostly causes asymptomatic infections in children as well as acute mild to severe hepatitis in adults, and this is followed by lifelong immunity. Acute hepatitis A has a relatively long incubation period (15 to 50 days). Fecal-oral transmission caused by contaminated drinking water, poor sanitation, and personal hygiene is by far the most frequent transmission route, and this is followed by oral-anal contact, as accounted only recently (1). A safe and effective vaccine can prevent hepatitis A (https://www.who.int/news-room/fact-sheets/detail/hepatitis-a).

For over four decades, the routine laboratory diagnosis of hepatitis A relied almost exclusively on serology, usually via the detection of anti-HAV IgM antibodies in blood and, rarely, seroconversion for anti-HAV IgG antibodies. Anti-HAV IgM antibodies are usually present at symptom onset, persist for 4 to 6 months, and are rarely detected a year after infection. Anti-HAV IgG antibodies appear almost concomitantly with anti-HAV IgM antibodies but persist lifelong in most recovered individuals as well as years after vaccination against HAV (https://www.ecdc.europa.eu/en/hepatitis-A/facts). Although anti-HAV IgM testing is quite reliable, several false-positive anti-HAV IgM results have been reported, thereby highlighting the need for the cautious interpretation of positive results, always in the context of patient history and other laboratory and clinical parameters, and especially in low-incidence settings (2–8).

HAV RNA can be detected in blood and stool as early as 2 weeks before anti-HAV antibodies, with a median positivity duration of 3 months (range 36 to 391 days) (9). Accordingly, HAV RNA screening was initially implemented in the pharmaceutical industry and in some high-income countries to ensure blood product safety. Although technically feasible for years, HAV RNA testing joined the diagnostic armament in routine virological laboratories mainly in the last decade. However, in many areas, its daily availability remains limited to reference or specialized laboratories. Namely, unlike high-volume anti-HAV serology, HAV RNA testing is rarely requested by clinicians, and this is usually after detecting anti-HAV IgM in patients with a strongly suspected false-positive anti-HAV IgM result, in individuals with negative and/or indeterminate anti-HAV IgM results when an early phase of acute hepatitis A is considered, or in severely immunocompromised patients (4, 10). For timely diagnosis and the avoidance of an additional blood draw, reflex HAV RNA testing is often performed using the same blood specimen that was initially sent for anti-HAV serology.

To the best of our knowledge, we present the first known report of HAV RNA PCR false-positive results observed during reflex HAV RNA testing and caused by the contamination of original blood samples with HAV RNA present in the reagents of the commercial anti-HAV immunoassay. The reason for occasional HAV RNA PCR false-positive results remained unresolved for 9 years, until it was linked with the preceding testing of serum samples for anti-HAV antibodies by one of the three automated immunoassay analyzers that was used in the laboratory.

## RESULTS

Our viral hepatitis reference laboratory started routine HAV RNA testing in 2005. Unlike frequent requests for anti-HAV serology, HAV RNA testing was requested about 18 times annually (range, 5 to 61 times), except for 2022, when, due to a HAV outbreak in Hungary, requests increased to a few hundred per year.

In 2012, our laboratory started observing sporadic HAV RNA-positive cases that were inconsistent with patients’ anti-HAV serological profiles and/or medical histories, suggesting that these cases could be associated with occasional laboratory contamination. Several measures, described in detail in Materials and Methods, were taken to identify the potential source of HAV RNA contamination that was leading to the sporadic HAV RNA false-positive results. All serum samples with suspicious initial false-positive HAV RNA results repeatedly tested HAV RNA-positive using three commercial PCR assays as well as an “in-house” HAV RNA PCR test, both in our laboratory and in other laboratories at our institution, including laboratories that had never dealt with hepatitis A positive samples. The Sanger sequencing of the PCR products clearly confirmed the presence of HAV RNA in all of the samples that had a suspicious initial false-positive HAV RNA result, from which HAV sequences were successfully generated.

However, the close follow-up of individuals that were initially anti-HAV IgM-negative (and HAV RNA “positive”) revealed that none seroconverted for anti-HAV antibodies in the following months. Similarly, all available follow-up serum and/or stool samples from individuals with suspected initial false-positive HAV RNA results clearly tested HAV RNA-negative. In addition, all of the PCR-contamination check samples that were collected from various laboratory surfaces, previously HAV-negative samples, blank water samples that were included in each PCR run, and serum samples that were obtained from nine healthy HAV RNA-negative individuals consistently tested HAV RNA PCR-negative.

In addition, the laboratory was 100% proficient in all HAV RNA EQA control panels purchased, and no single HAV RNA false-positive result was detected when testing the HAV RNA-negative EQA panel samples.

Finally, close inspection and a detailed step-by-step review of all of the testing protocols that were used in our laboratory revealed a correlation between the HAV RNA false positivity and the preceding testing of serum samples with Elecsys Anti-HAV or Elecsys Anti-HAV IgM assays on a Cobas e411 automated immunoassay analyzer (Roche Diagnostics, Mannheim, Germany), which is one of the three immunoassay analyzers that was used in our laboratory from 2012 to 2018. The preceding testing of serum samples for anti-HAV IgG and/or anti-HAV IgM antibodies with two other automated immunoassay analyzers that were in use, namely, a Vitros 3600 (Ortho Clinical Diagnostics, Illkirch, France) and an ARCHITECT i2000SR (Abbott Laboratories, Wiesbaden, Germany), did not result in subsequent HAV RNA false positivity during reflex HAV RNA testing. Therefore, the HAV RNA contamination of samples during the preceding testing for anti-HAV antibodies on the Cobas e411 was strongly suspected as a source of the sporadic HAV RNA PCR false-positive results that were recorded in our laboratory during the nine-year period.

**FIG 1:**
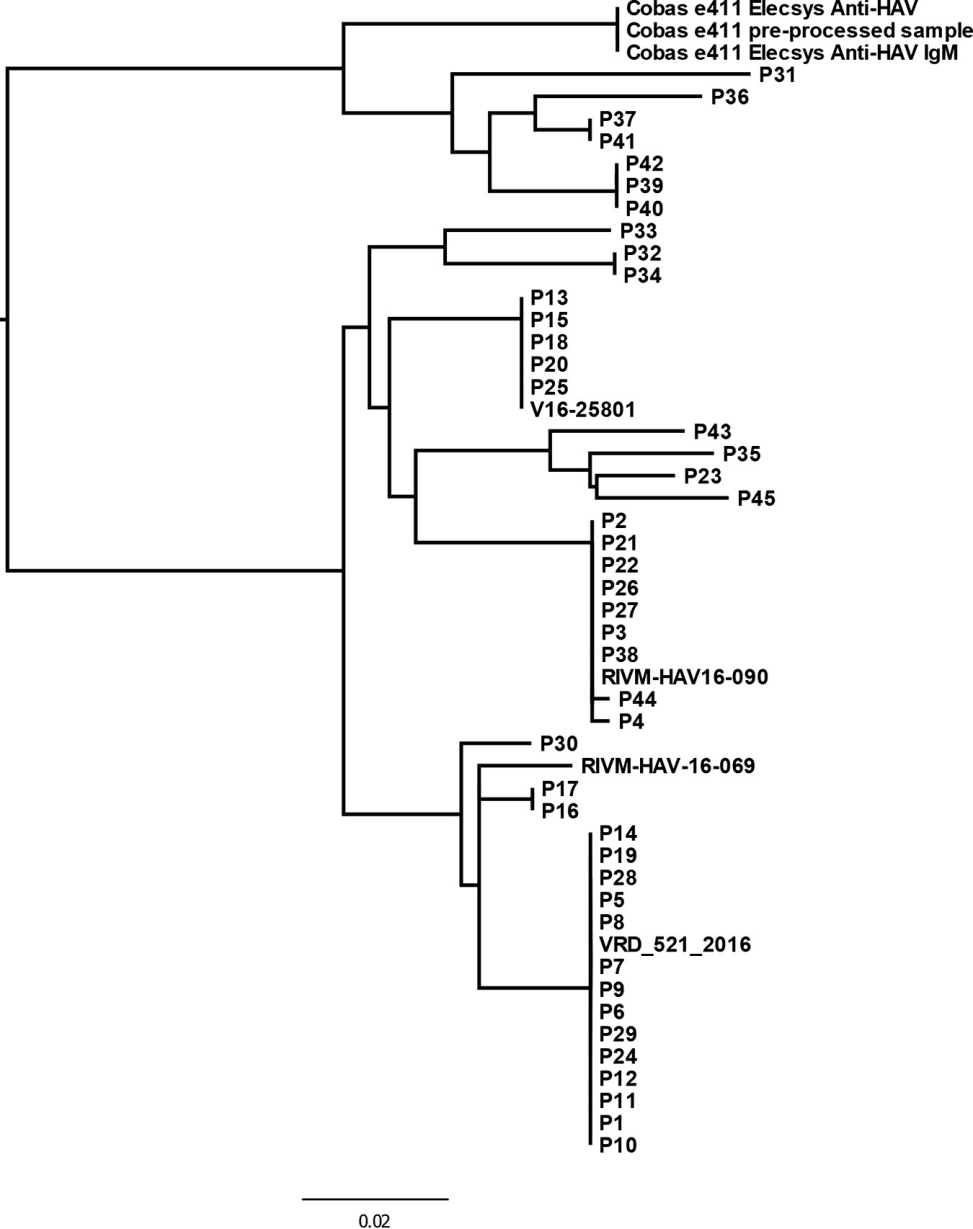
Maximum likelihood phylogenetic tree of HAV sequences obtained from Cobas e411 Elecsys anti-HAV and anti-HAV IgM reagents, the serum sample preprocessed on a Cobas e411, the available sequences from Slovenian patients with confirmed HAV infections between 2012 and 2020 as well as the reference sequences of major ECDC-reported HAV outbreaks (V16-25801, RIVM-HAV16-090, RIVM-HAV-16-069, and VRD_521_2016) during this time frame.

To confirm our hypothesis, we first tested four serum samples from four healthy individuals for the presence of HAV RNA, and all four tested HAV RNA-negative. Then, the same four samples were first tested on a Cobas e411 for the presence of anti-HAV antibodies using Elecsys Anti-HAV and Elecsys Anti-HAV IgM assays, and this was followed by reflex HAV RNA testing. All four samples that had been previously processed on a Cobas e411 clearly tested HAV RNA-positive, with the cycle threshold (CT) values ranging from 27 to 29. The presence of HAV RNA was also consistently detected in different batches of reagents of both the Cobas e411 Elecsys Anti-HAV and Anti-HAV IgM assays, with the CT values ranging from 20 to 24. HAV sequences were obtained from all four of the Cobas e411 anti-HAV preprocessed samples as well as directly from the Cobas e411 anti-HAV reagents, and they showed 100% identity. Furthermore, a phylogenetic analysis showed that these sequences were significantly different from all of the HAV sequences that were generated from patients with clearly confirmed HAV infections in Slovenia between 2012 and 2020 (Fig. 1).

Interestingly, although the presence of HAV RNA was also consistently detected in different batches of anti-HAV reagents from the two other automated immunoassay analyzers that were used in our laboratory (Vitros 3600 and ARCHITECT), due to the different pipetting modes of these two analyzers in comparison to the Cobas e411, HAV RNA false positivity was never detected in the HAV RNA reflex testing of samples that were preprocessed in either the Vitros 3600 or the ARCHITECT.

## DISCUSSION

Reflex molecular testing following serological screening/testing is a frequent and beneficial practice in virological laboratories that offer HIV and hepatitis testing services. Preferably, reflex molecular testing should be performed on a different aliquot of a sample than the one that was used for (preceding) serological testing or from another sample that has been concomitantly received from the same patient. Such a protocol has been a standard practice for years for reflex testing in our laboratory for HIV and hepatitis B and C, and, in recent years, also for hepatitis A, D, and E. However, when an unpredicted positive or intermediate serological test result is obtained and reflex molecular testing is not initially considered, as well as in the case of a limited quantity of available specimen, to ensure timely diagnosis and to avoid an additional blood draw, reflex molecular testing is also performed on samples that have been preprocessed in immunoassay analyzers. In most cases, such a practice is absolutely valid and beneficial, but, exceptionally, as described here, it could be risky and could have significant clinical consequences.

To the best of our knowledge, this is the first report of HAV RNA false-positive results that have been detected during reflex HAV RNA testing and caused by HAV RNA that was present in the reagents of a commercial anti-HAV immunoassay. HAV RNA was consistently detected in different batches of the anti-HAV reagents of all of the three automated immunoassay analyzers that were in use in our laboratory (Cobas e411, Vitros 3600, ARCHITECT), most likely due to the presence of the lysates of the inactivated (whole) viruses (containing traces of viral RNA) that were used as antigens in the anti-HAV reagents instead of recombinant HAV antigens (containing no viral RNA). However, only the use of one analyzer and the corresponding commercial anti-HAV immunoassay reagents resulted in contamination that led to false positive HAV RNA results. The principal reason for the false positive HAV RNA results was a peculiar pipetting mode of action of the Cobas e411, in which the analyzer uses a single pipette tip to first collect the anti-HAV reagent and then reuses the same pipette tip to collect the patient’s serum/plasma sample, which consequently causes the permanent contamination of the original sample with HAV RNA. In contrast, both the Vitros 3600 and the ARCHITECT use separate pipette tips to collect anti-HAV reagents and the sample. Such a unique and potentially harmful Cobas e411 pipetting approach is not disclosed in the instrument’s manual, and, consequently, for years, the laboratory was unaware of this important instrument drawback. The main reason that occasional HAV RNA false-positive results were not encountered earlier is that between 2012 and 2020, the Cobas e411 was used only as a backup analyzer, mainly for the resolution of initially indeterminate anti-HAV Vitros 3600 results, and, consequently, only 1 to 3 patients per year had HAV RNA-positive results that were inconsistent with patients’ anti-HAV serological profiles and/or medical histories. In late 2020, when the Cobas e411 become the frontline analyzer for anti-HAV serology, the number of suspicious HAV RNA false-positive results increased substantially to 10 per month, prompting immediate close inspection and a detailed, step-by-step review of all of the testing protocols that were being used in our laboratory. This finally resulted in the identification of the source and mode of the HAV RNA contamination that was causing the occasional HAV RNA false-positive results. Finally, we stopped using the Cobas e411 for anti-HAV serology in October of 2020.

In conclusion, we describe occasional HAV RNA false-positive results that were detected during reflex molecular testing and linked with the preceding testing of serum samples for anti-HAV antibodies using an automated immunoassay analyzer. The peculiar pipetting procedure of this analyzer with a single pipette tip to collect both anti-HAV reagents and the sample contaminated the original patient sample with the HAV RNA that was present in the immunoassay’s reagents, thereby resulting in HAV RNA false positivity during reflex molecular testing. Manufacturers of automated immunoassay analyzers and corresponding assays should strongly consider the occasional need for reflex testing from preprocessed samples and design their products to prevent the contamination of processed samples, either by using separate pipette tips when collecting reagents and samples or by using recombinant viral proteins instead of inactivated whole viruses (containing traces of viral RNA or DNA) as antigens in their immunoassays (11). If a testing procedure is prone to the contamination of the original sample with the viral RNA/DNA that is present in the assay’s reagents, the manufacturer should state this clearly in the package insert and advise against reflex molecular testing from preprocessed samples. Reflex molecular testing should preferably be performed from a separate aliquot of the original sample or from a separate concomitant sample. The reflex testing of samples that are preprocessed in an automated immunoassay analyzer should be performed with great care.

## MATERIALS AND METHODS

The following measures were taken to identify the potential source of the HAV RNA contamination that was leading to sporadic HAV RNA false-positive results: (i) all questionable samples were tested for HAV RNA in parallel using several commercial PCR assays, namely, the artus HAV RT-PCR Kit (Qiagen, Hilden, Germany), then LightCycler HAV Quantification Kit (Roche Diagnostics), and the AltoStar HAV RT-PCR Kit (Altona Diagnostics, Hamburg, Germany), strictly following the manufacturers’ instructions, as well as the “in-house” HAV RNA PCR test (12); (ii) HAV RNA testing was performed in different laboratories at our institution, including those that had never dealt with hepatitis A-positive samples; (iii) all suspicious HAV RNA-positive samples were Sanger sequenced and typed according to the HAVNET protocol (https://www.rivm.nl/en/havnet); (iv) additional follow-up serum and/or stool samples were collected from all individuals with suspected false-positive HAV RNA results; (v) frequent PCR-contamination checks of various laboratory surfaces were performed; (vi) several previously HAV RNA-negative samples and blank water samples were included in each PCR run; (vii) several serum samples were obtained from healthy anti-HAV and HAV RNA-negative controls and tested for HAV RNA; and (viii) multiple rounds of HAV RNA external quality assessment (EQA) control panels (Hepatitis A Virus Mini-Panel 01 [Qnostics] and QCMD Hepatitis A virus RNA EQA Program 2012, 2019 to 2022) were purchased and tested for HAV RNA.

A phylogenetic tree was inferred from all of the laboratory-generated HAV sequences from 2012 to 2020 using PhyML 3.0, with model selection occurring in accordance with the Bayesian information criterion (13).
